# Biomedical event extraction based on GRU integrating attention mechanism

**DOI:** 10.1186/s12859-018-2275-2

**Published:** 2018-08-13

**Authors:** Lishuang Li, Jia Wan, Jieqiong Zheng, Jian Wang

**Affiliations:** School of Computer Science and Technology, Dalian University of Technology, Dalian, China

**Keywords:** Biomedical event extraction, Attention mechanism, Word representation, Deep learning

## Abstract

**Background:**

Biomedical event extraction is a crucial task in biomedical text mining. As the primary forum for international evaluation of different biomedical event extraction technologies, BioNLP Shared Task represents a trend in biomedical text mining toward fine-grained information extraction (IE). The fourth series of BioNLP Shared Task in 2016 (BioNLP-ST’16) proposed three tasks, in which the Bacteria Biotope event extraction (BB) task has been put forward in the earlier BioNLP-ST. Deep learning methods provide an effective way to automatically extract more complex features and achieve notable results in various natural language processing tasks.

**Results:**

The experimental results show that the presented approach can achieve an F-score of 57.42% in the test set, which outperforms previous state-of-the-art official submissions to BioNLP-ST 2016.

**Conclusions:**

In this paper, we propose a novel Gated Recurrent Unit Networks framework integrating attention mechanism for extracting biomedical events between biotope and bacteria from biomedical literature, utilizing the corpus from the BioNLP’16 Shared Task on Bacteria Biotope task. The experimental results demonstrate the potential and effectiveness of the proposed framework.

## Background

With the rapid development of computational and biological technology, biomedical literatures are expanding at an exponential rate, which makes it difficult to extract the required information by hand and also provides an opportunity for text mining techniques in this field. In past years, the major focus of biomedical text mining has been named entity recognition (NER), which identifies entities such as genes, proteins and drugs. Recently, text mining researchers pay more attention to complex information extraction, such as biomedical event extraction, with the appearance of applicable NER systems.

**Fig. 1:**
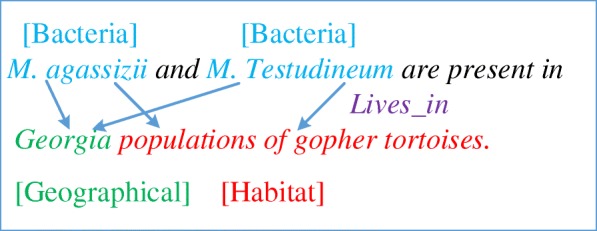
Example of entities and Lives in events in the BB task. “Georgia” is the entity of [Geographical]; “populations of gopher tortoises” is the entity of [Habitat]; “*M. agassizii*” and “M. Testudineum” are the entities of [Bacteria]. There is relation of “Live in” between the bacterium entity and a Habitat or a Geographical entity

As the primary forum for international evaluation of different biomedical event extraction technologies, BioNLP Shared Task represents a trend in biomedical text mining toward fine-grained information extraction (IE). The fourth series of BioNLP Shared Task in 2016 (BioNLP-ST’16) proposed three tasks, in which the Bacteria Biotope event extraction (BB) task has been put forward in the earlier BioNLP-ST. BB task focus on studying the interaction mechanisms of the bacteria with their environment from genetic, phylogenetic and ecology perspectives. The BB task involves three types of entities, Bacteria, Habitats and Geographical places. It also involves a single type of event, the Lives in event, which is a relation between two mandatory arguments, the bacterium and the location where it lives, either a Habitat or a Geographical entity. Fig. 1 displays an example of entities and events in the BB task.

Previous BB tasks have been raised in 2011 and 2013 with similar subtasks. Ratkovik et al.’s framework based on semantic analysis supported by an ontology of the biotope domain achieved the first place in the BioNLP-ST’11 [1]. After the BioNLP-ST’11, the BB task in the BioNLP-ST’13 introduced a same event extraction task on a new dataset [2]. Björne et al.’s system was the best system in BioNLP-ST’13, which adopted support vector machine (SVM) and extracted a wide array of features based on dependency parse graphs, achieving an F-score of 42% on the test datasets [3]. In the BioNLP-ST’16, team VERSE obtaining the 1st place in BB-event subtask utilized SVM and achieved an F-score of 55.8% [4]. The methods mentioned above for BB event extraction are based on shallow machine learning methods, which utilized commonly used features and achieved good results.

Recently, deep learning methods provide an effective way to automatically extract more complex features and achieve notable results in various natural language processing (NLP) tasks. Zhang proposed a bidirectional long short-term memory networks (BLSTM) to model the sentence with complete, sequential information about all words for relation classification [5]. Jagannatha adopted original sentences as the input of LSTM and GRU, and the results showed that LSTM and GRU are valuable tools for extracting medical events in the Electronic Health Record (EHR) notes [6]. Team TurkuNLP used a combination of several LSTM networks over syntactic dependency graphs, which achieved an F-score of 52.1% in BB-event subtask in the BioNLP-ST’16 [7]. Team DUTIR employed convolutional neural network (CNN) to model the sentences by convolution and maxpooling operation from raw input with word embeddings and used fully connected neural network to learn senior and significant features automatically, which reached 47.8% F-score [8]. Li proposed a bidirectional LSTM-based recurrent neural network and a dynamic extended tree was introduced as the input, which achieved an F-score of 57.14% [9]. After that, Li utilized the predictions of SVM for post-processing, which reached 58.09% F-score [10]. However, all words are equally important in these approaches, which leads to the failure to capture the most important semantic information in a sentence.

Attention mechanism has been introduced to the NLP task to capture the semantic attention and promote the performance of a variety of task, which has recently succeeded in a variety of tasks ranging from machine translations [11], speech recognition [12], to image captioning [13]. Zhou et al. [14] presented an attention-based bilingual representation learning model which learned the documents in both the source and the target languages and a hierarchical attention mechanism for the bilingual LSTM network. Zhou et al. [15] proposed Attention-Based Bidirectional LSTM to handle the relation classification on the SemEval-2010 task. In this paper, we propose an attention-based BGRU (Bidirectional Gated Recurrent Unit) network architecture for the Bacteria Biotope event extraction. The BGRU networks as a deep learning framework can reduce the number of handcrafted features and overcome the problems above. Furthermore, the attention mechanism can take advantage of the important information in the sentence and make the semantic information more precise.

Deep-learning methods are representation-learning methods, by which deep learning framework can perform well. Mikolov et al. [16, 17] proposed two efficient models, continuous bag-of-words model (CBOW) and skip-gram model, to learn word embeddings from large-scale text corpora. The above models are designed with general-purpose, usually trained on Wikipedia and evaluated on word analogy tasks. However, the experimental evidence shows that domain irrelevant word embeddings trained on large collections of texts are not good enough for biomedical domain NLP, biomedical-oriented word embeddings can outperform general-purposed ones, and further improve the performance of biomedical NLP systems. Therefore, we utilize a biomedical domain-specific word representation model, which integrates biomedical information into word embeddings.

In conclusion, the main contributions of this study are summarized as follows:

- We propose an attention-based BGRU network architecture for the Bacteria Biotope event extraction. Compared to the previous methods which cannot capture the important information, the attention mechanism has obvious advantage to put greater weight to the useful information, which enables our model to focus on certain part of the input. In addition, the deep learning method used in our model, BGRU networks, avoids extracting the manual features.
- A domain-oriented word representation is employed in our model. Because biomedical text has many domain-specific features that should be taken into consideration, such as special characters in gene sequences and widely existed biomedical entities, we consider biomedical text as a sequence of words, syntactic chunks and biomedical entities and incorporate them into word embeddings.

The remaining part of the paper is organized as follows. Section II detailed illustrates the proposed method. Section III presents the experimental results. Section IV and section V discusses and concludes our paper.

## Methods

**Fig. 2:**
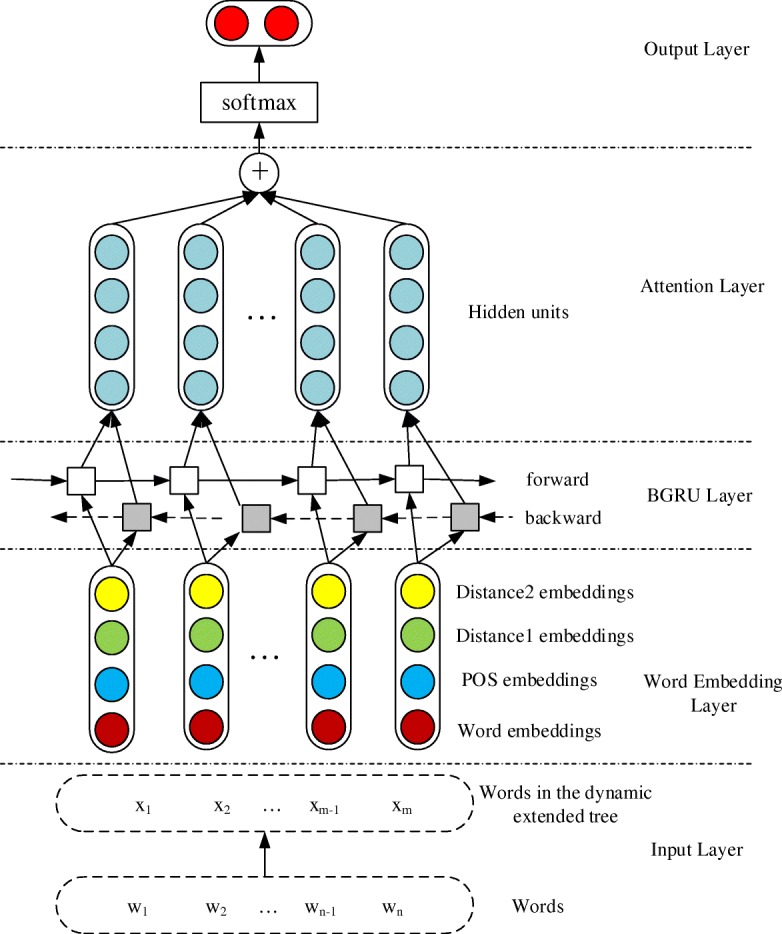
The architecture of attention-based BGRU

We explore an attention-based BGRU network architecture for the Bacteria Biotope event extraction, in which the attention mechanism and distributed representation for biomedical domain are introduced. The system framework for event extraction can be generalized in Fig. 2. As shown in Fig. 2, the model proposed in this paper contains five components and we will take the instance “The effects of drinking a fermented milk beverage that contains Lactobacillus casei strain Shirota (LcS) at 40 billion bacterial cells/bottle for 4 weeks (probiotics, 1 bottle/day) on defecation frequency, intestinal microbiota and the intestinal environment of healthy individuals with soft stools were evaluated.” to demonstrate our work:

1. Input layer: After the replacement of two entities, the sentence become “The effects of drinking a fermented milk beverage that contains Lactobacillus casei strain entity_1 ( LcS) at 40 billion bacterial cells / bottle for 4 weeks ( probiotics , 1 bottle / day) on defecation frequency , intestinal microbiota and the intestinal environment of healthy individuals with soft entity_2 were evaluated.” . We obtain the Shortest Path enclosed Tree (SPT) between two entities by GENIA Dependency parser (GDep) [18] to get the informative words. So the instance becomes “(ROOT (FRAG (NP (NNP entity_1)) (PP (IN at) (NP (NN environment))) (PP (IN with) (NP (NNP entity_2)))))”. In order to obtain more information, the SPT is extended to the dynamic extended tree (DET) [19]. Therefore, we can get the instance “(ROOT (SINV (VP (VBZ contains)) (NP (NP (NNP entity_1)) (PP (IN at) (NP (NP (NN environment)) (PP (IN with) (NP (NNP entity_2))))))))”.
2. Embedding layer: Every word in the input is represented by concatenating the word embeddings, POS embeddings and distance embeddings. The word embeddings and Part-of-speech (POS) embeddings are trained through domain-oriented word representation model in advance, while the distance embeddings are initialized by Zeng’s method [20]. Thus the DET is mapped to embeddings.
3. BGRU layer: The hidden layer is acquired by a recurrent neural network with attention-based BGRU. BGRU networks pick up the information from forwards and backwards of the sentence respectively.
4. Attention layer: A weight vector could learn word features automatically and record the significant information in a sentence. A sentence feature can be represented by multiplying the weight vector.
5. Output layer: We predict the label for classification by a softmax function. So we can predict whether there is an interaction between the entities “entity_1” and “entity_2”.

In the following sections, we present the implementation for these stages in our biomedical event extraction system in detail and evaluate our system on the BioNLP-ST’16 BB-event datasets.

### A. Word representation

Distributed representations of words in a vector space have achieved great success in NLP tasks by grouping similar words. The basic idea of distributed representation is that vectors are trained by surrounding words. However, many biomedical entities and syntactic chunks, which contain rich domain information in biomedical text mining, are not considered. In this paper, we utilize our domain-oriented word representation model to train word embeddings, which incorporates domain information such as biomedical entities. The dataset downloaded from PubMed is used to train word embeddings.

Firstly, we use GDep which is more suitable for biomedical corpus to extract the following biomedical information including stem, chunk, entity and part-of-speech tags.

#### Stem

We take the input sentence “contains [bacterium]_e1_ at environment with [human]_e2_” as an example. Although we all know that “contains” is the plural form of “contain”, machines do not realize it. Instead, machines regard “contain” and “contains” as two totally different tokens. Considering stem can solve the problem.

#### Chunk

Only considering words and stems may be not enough. If there are modifiers in front of “bacterium”, all modifiers of “bacterium” should be considered as a whole. Therefore, considering syntactic chunks is essential.

#### Entity

Entity is the result of named entity recognition, which provides information on biological entities and fine-grained understanding of biomedical text.

#### POS tags

POS reflects the role of words in a sentence, which is important for the analysis of sentence structure. So we use the POS to train embeddings for gaining more information.

Then embeddings are trained by surrounding words and their biomedical information extracted by GDep.

Finally, we can acquire word embeddings and POS embeddings.

In our system, three types of embeddings are concatenated to compose our representation of the input, whose dimension is defined as *d*^*w*^. Three types of embeddings are described as follows:

#### Word embeddings and POS embeddings

In our experiment, word embeddings are trained by domain-oriented word representation. In addition, our POS tags are parsed in NLTK parser which is a coarse-grained POS category. e.g., “pos:NN”, etc. The training method of POS embeddings is similar to the word embeddings and both of them are obtained by lookup tables.

#### Distance embeddings

According to the corpus analysis, we find that the entity pair is more likely to constitute an event if the distance (the number of words) between the two entities is short. For this purpose, Zeng et al. [20] proposed the use of distance embeddings (position features) which helped the CNN by the distance embeddings. In this work we also experiment with the distance embeddings. The distance embeddings are derived from the relative distances of the current word to the target “bacterium” and “human”. For instance, in the sentence shown above, the relative distances of “at” to “bacterium” and “human” are “-1” and “3”, respectively. Each relative distance is mapped to a vector of settled dimension. Each dimension *d(l)* of the distance embeddings is initialized with eq. (1),

1 $$ d(l)=\tanh \left(l/s\right) $$

where the relative distance is *l*. *s* refers to the maximum of the relative distances in the corpus. *d(l)* is copied many times, which are equal to settled dimension. Suppose the vectors *d*_*1*_ and *d*_*2*_ represent the distance embeddings of the current word to the targets “bacterium” and “human” respectively, the distance embeddings *d*_*t*_ of the current word *w* is given by the concatenation of these two vectors, *d*_*t*_ *=* [*d*_*1*_*;d*_*2*_].

### B. GRU

**Fig. 3:**
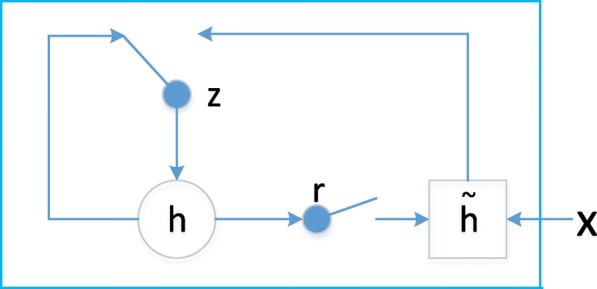
Hidden activation function of GRU

The gated recurrent unit (GRU) [21] adaptively remembers and forgets its state based on the input signal to the unit. We also explore the more complex LSTM but it performs similarly and is more computationally expensive. A standard architecture of GRU is shown in Fig. 3.

We will describe how the activation of the *j-th* hidden unit is computed. Firstly, at time-step *t*, the GRU units can ignore whenever the detected feature is not necessary anymore considering the previous hidden states and the current input by using the reset mechanism with eq. (2).

2 $$ {r}_j=\sigma \left({\left[{W}_rx\right]}_j+{\left[{U}_r{h}_{\left\langle t-1\right\rangle}\right]}_j\right), $$

where *σ* is the logistic sigmoid function, and [.]_*j*_ denotes the *j-th* element of a vector. *h*_*t-1*_ is the state of the previous step and *x* is the current input. Then the new candidate memory content ${\tilde{h}}_t$ is computed considering the reset gate *r*_*j*_ with eq. (3).

3 $$ {{\tilde{h}}^{\left\langle t\right\rangle}}_j=\tanh \left({\left[ Wx\right]}_j+{\left[U\left(r\odot {h}_{\left\langle t-1\right\rangle}\right)\right]}_j\right), $$

where ⊙ is an element-wise multiplication. Then the update gate *z*_*j*_ controls how much of the previous memory content is to be forgotten and how much of the new memory content is to be added with eq. (4):

4 $$ {z}_j=\sigma \left({\left[{W}_zx\right]}_j+{\left[{U}_z{h}_{\left\langle t-1\right\rangle}\right]}_j\right). $$

Finally, the new memory state is obtained through the update mechanism as eq. (5).

5 $$ {h}_j^{\left\langle t\right\rangle }={z}_j{h}_j^{\left\langle t-1\right\rangle }+\left(1-{z}_j\right){\tilde{h}}_j^{\left\langle t\right\rangle }, $$

where *W*_*r*_*, U*_*r*_*, W, U, W*_*z*_*, U*_*z*_ are weight matrices which are learned.

### C. BGRU

One shortcoming of unidirectional GRU is that it is only able to utilize the previous context. BGRU can solve this by processing the data in both directions with two separate hidden layers, which are then fed forwards to the same output layer. As shown in Fig. 2, the BGRU network contains two sub-networks for the left and right sequence context. Let *D* be a matrix consisting of output vectors [*h*_*1*_*, h*_*2*_*, ..., h*_*n*_] that the final hidden state of the forward GRU and the backward GRU produced, where $D\in {\mathrm{\mathbb{R}}}^{d^w\times n}$ and *n* is the sentence length. The outputs of these subnets for the word at time *t* are integrated in the following way with eq. (6):

6 $$ D=\frac{D_f^{\left\langle t\right\rangle }+{D}_b^{\left\langle t\right\rangle }}{2}, $$

where *f* and *b* refer to the forward and backward directions respectively.

### D. Attention

According to the analysis of corpus, different words in a sentence usually have different influence in the overall semantic information. Some words can be decisive while the others are irrelevant. Thus we try to find the important units in the sequence influential for the output, which can be achieved by attention mechanism.

In this study, we propose the attention mechanism for BB event extraction. We use attention mechanism that assigns greater weight to the more important words, which makes the semantic information be fully used. For a sentence including *n* words, *w*_*i*_ and *m*_*i*_ denote *i-th* word and its attention weight respectively. Attention weights are normalized which satisfy the eq. (7):

7 $$ \sum \limits_i{m}_i=1. $$

Traditional GRU based models represent the word sequences only using the hidden layer *D* at the final node. However, in our model, the hidden states at all the positions are considered with different attention weights in order to focus on some certain parts of the sentence and filter out the irrelevant semantic noise.

Firstly, we utilize the activation function tanh to handle the final state *D* of the GRU with eq. (8):

8 $$ H=\tanh (D). $$

Then, we introduce parameter vector *p* which is randomly initialized. The vector’s transpose is *p*^*T*^ and the dimension is *d*_*w*_ to train and predict the attention weights with eqs. (9) and (10):

9 $$ \widehat{m}={p}^TH, $$

10 $$ {m}_i=\frac{\exp \left({\widehat{m}}_i\right)}{\sum \limits_i\exp \left({\widehat{m}}_i\right)} $$

Let *α* be a vector consisting of numbers [*m*_*1*_*, m*_*2*_*, …, m*_*n*_], called attention vector.

The representation *r* of the sentence is formed by a weighted sum of these output vectors (11):

11 $$ r=D{\alpha}^T, $$

where the dimension of *r* is *d*^*w*^.

At last, we obtain the overall semantic information of the sentence used for classification from eq. (12):

12 $$ o=\tanh (r) $$

## Results

### A. Corpus and evaluation

The dataset is provided by the Bacteria Biotope event extraction task of BioNLP-ST’16, which is a subset of a corpus of 1.16 million PubMed references and the manual annotation of bacteria and habitats was performed by Bibliome group. In our work, we only consider relations within sentences.

All experiments are evaluated with online evaluation. The evaluation method is the matching similarity. Namely, if the Bacteria argument in the reference and the prediction events are the same entity or equivalent entities, and the Location argument in the reference and the prediction events are the same entity or equivalent entities then the matching similarity is 1, otherwise 0. The results are measured using Recall, Precision and F-score. The definition of Precision (P), Recall (R) and F-score (F) are shown as eq. (13):

13 $$ P=\frac{TP}{TP+ FP},R=\frac{TP}{TP+ FN},F- score=\frac{2\ast P\ast R}{P+R}. $$

where *TP* is short for true positives, *FP* represents false positives, and *FN* stands for false negatives. Any cross-sentence relations in the test data count against the submission as false negatives.

### B. Hyper parameters and training details

This subsection presents the hyper parameter tuning for our model. The dimensions of word embeddings, POS embeddings and distance embeddings are set as 50. We use unlabeled corpus downloaded from PubMed to train word representation, which consists of 5.99 million words. The initial word embeddings are trained by setting the windows to be 5 and learning rate to be 0.025. The dropout rate is set to be 0.5 to prevent overfitting. As it is not feasible to perform a full grid search for all hyper parameters, the above values are chosen empirically. The existing training/dev split for the data is used in the paper. Referring to Mehryary’s [7] discussion with regard to the challenge of deep learning methods on a small test set, where the optimal length of training is four epochs by the experiment, the number of iterations in our model is set between three and five. The “learning rate” is set to 10^− 3^, which is chosen by validation from the set {10^− 2^, 10^− 3^, 10^− 4^}. The dropout method is tried to apply on the hidden state of the forward GRU, the hidden state of the backward GRU and the final hidden state of the GRU. Finally, the dropout method is applied on the final hidden state of the GRU. The “batch_size” is set to 5 and the number of layers is 1. The parameters mentioned above are set from development set. We implement the framework based on Theano [22] and use a GTX TITAN graphic card for training.

### C. Baselines and results

Table 1 compares the performance among our system, the baseline and other excellent systems. The baseline was implemented based on Björne al.’s (2013) paper. Compared to baseline, the precision of our system is not better than the baseline, however, the recall is 31.47 percentage points higher than the baseline. From Table 1 we can see that the F-score of our system is 10.15 percentage points higher than the baseline system. The reason is that the Björne’s system was characterized by a wide array of features based on dependency parse graphs, therefore it achieved the higher precision. However, the recall was not satisfactory and it illustrates that excessive manual efforts may hurt the generalization performance.

**Table 1:** Comparison with existing systems

Methods	F-score	Recall	Precision
Baseline	47.27%	38.35%	61.61%
TurkuNLP	52.10%	44.80%	62.30%
VERSE	55.80%	61.50%	51.00%
Li [9]	57.14%	57.99%	56.32%
Li [10]	58.09%	56.80%	59.44%
BGRU-Attention	57.42%	69.82%	48.76%

The best system in BioNLP-ST’16 is the VERSE, obtaining the 1st place in BB-event subtask, achieved an F-score of 55.8%. VERSE offered control on the exact semantic features to use for classification and allowed feature selection to reduce the size of feature vectors. Although our system is only 1.62 percentage points higher than it, we need not spend a lot of energy to select handcrafted features.

Team TurkuNLP used a combination of several LSTM networks over syntactic dependency graphs, which achieved 52.1% F-score and obtained the 2nd place in BB-event subtask in the BioNLP-ST’16. Li [9] proposed a bidirectional LSTM-based recurrent neural network and used a dynamic extended tree as the input, which achieved an F-score of 57.14%. Li’s [10] method outperforms ours because their system used two classifiers and improved the precision. However, their model treated all words equally and could not capture the decisive information. Our approach integrates attention mechanism, which gives different words different weights. So, the important words are emphasized. The results indicate the effectiveness of our method.

### D. Influence of the attention mechanism

In this study, we propose an attention mechanism to capture the related semantic information of each word. In Table 2, we show the results of models with attention mechanisms. The models are based on the bi-directional GRU network as shown in Fig. 2. BGRU is the basic bi-directional GRU network without attention mechanism. From the results, we can observe that BGRU+Attention outperforms well, whose F-score is 1.06 percentage points higher than the BGRU model, which proves the effectiveness of the attention mechanism.

**Table 2:** The results of the attention mechanism

Input	F-score	Recall	Precision
BGRU	56.36%	63.42%	50.71%
BGRU+Attention	57.42%	69.82%	48.76%

### E. Influence of the word Embeddings

For the deep learning methods, the initial word embeddings used as the inputs for the network usually play an important role in models. We compare the domain-oriented method and the skip-gram model to train word embeddings. All parameters described in the previous section are set the same. The performance on the BB-event task for two kinds of word vectors are shown in Table 3. From the results, we can see that the F-score of our domain-oriented method is 1.45 percentage points higher than the skip-gram model, which proves that incorporating biomedical information is beneficial to embeddings.

**Table 3:** The results of the word embeddings

Word representation	F-score	Recall	Precision
skip-gram	55.97%	62.43%	50.72%
domain-oriented method	57.42%	69.82%	48.76%

## Discussion

From the above experimental results, we can conclude that our BGRU model based on attention mechanism performs well and mainly includes the following important advantages:

### No complex hand-designed features and using domain-oriented word embeddings

We skip the step of extracting complex hand-designed features, only adopt simple POS and distance features, and replace it with word embeddings trained beforehand. Since the *domain-oriented* word embeddings can catch a large number of syntactic and semantic word relationships and also capture biomedical information, the deep learning architecture can fully utilize them and extract the high-level features for the BB-event systems.

### Attention mechanism

All hidden states are treated equally before using an attention mechanism, which is not reasonable. Some words in a sentence play a decisive role, thus a higher weight should be given to make judgments more accurate, which can be well implemented by an attention mechanism. The experimental results also reveal that our attention mechanism is effective.

### Effective GRU framework

The results show that our GRU framework is effective in features detection and propagation. This is mainly because that the proposed attention mechanism, our selected embeddings and structure of GRU are suitable for BB-event.

## Conclusions

In this paper, we propose an attention-based BGRU network architecture for the Bacteria Biotope event extraction. We improve the model by utilizing attention mechanism. Simultaneously, the word embeddings for biomedical domain are added into the bidirectional GRU to obtain more abundant contextual information. The experimental results show that our model on BB-event corpus can achieve an F-score of 57.42% without using complex hand-designed features and external resource, which is 1.62 percentage points higher than the best systems. The experimental results demonstrate the potential and effectiveness of the proposed framework. However, there are still spaces for improvement. For example, coreference resolution can be used to find more events.

## Abbreviations

BB: Bacteria Biotope event extraction
BLSTM: Bidirectional long short-term memory net-works
CBOW: Continuous bag-of-words model
CNN: Convolutional neural network
FN: False negatives
FP: False positives
GDep: GENIA Dependency parser
GRU: Gated Recurrent Unit
EHR: Electronic Health Record
IE: Information extraction
NER: Named entity recognition
NLP: Natural language processing
POS: Part-of-speech
SPT: Shortest Path enclosed Tree
SVM: Support vector machine
TP: True positives
